# Safety and Oncological Outcomes of Laparoscopic NOSE Surgery Compared With Conventional Laparoscopic Surgery for Colorectal Diseases: A Meta-Analysis

**DOI:** 10.3389/fonc.2019.00597

**Published:** 2019-07-03

**Authors:** Rui-Ji Liu, Chun-Dong Zhang, Yu-Chen Fan, Jun-Peng Pei, Cheng Zhang, Dong-Qiu Dai

**Affiliations:** ^1^Department of Gastrointestinal Surgery, The Fourth Affiliated Hospital of China Medical University, Shenyang, China; ^2^Department of Gastrointestinal Surgery, Graduate School of Medicine, University of Tokyo, Tokyo, Japan; ^3^Cancer Center, The Fourth Affiliated Hospital of China Medical University, Shenyang, China

**Keywords:** natural orifice specimen extraction, colorectal diseases, oncological outcomes, post-operative function, totally intra-abdominal laparoscopic surgery, meta-analysis

## Abstract

**Objective:** To evaluate the safety and oncological outcomes of laparoscopic colorectal surgery using natural orifice specimen extraction (NOSE) compared with conventional laparoscopic (CL) colorectal surgery in patients with colorectal diseases.

**Methods:** We conducted a systematic search of PubMed, EMBASE, and Cochrane databases for randomized controlled trials (RCTs), prospective non-randomized trials and retrospective trials up to September 1, 2018, and used 5-year disease-free survival (DFS), lymph node harvest, surgical site infection (SSI), anastomotic leakage, and intra-abdominal abscess as the main endpoints. Subgroup analyses were conducted according to the different study types [RCT and NRCT (non-randomized controlled trial)]. A sensitivity analysis was carried out to evaluate the reliability of the outcomes. RevMan5.3 software was used for statistical analysis.

**Results:** Fourteen studies were included (two RCTs, seven retrospective trials and five prospective non-randomized trials) involving a total of 1,435 patients. Compared with CL surgery, the NOSE technique resulted in a shorter hospital stay, shorter time to first flatus, less post-operative pain, and fewer SSIs and total perioperative complications. Anastomotic leakage, blood loss, and intra-abdominal abscess did not differ between the two groups, while operation time was longer in the NOSE group. Oncological outcomes such as proximal margin [weighted mean difference [WMD] = 0.47; 95% confidence interval [CI] −0.49 to 1.42; *P* = 0.34], distal margin (WMD= −0.11; 95% CI −0.66 to 0.45; *P* = 0.70), lymph node harvest (WMD = −0.97; 95% CI −1.97 to 0.03; *P* = 0.06) and 5-year DFS (hazard ratio = 0.84; 95% CI 0.54–1.31; *P* = 0.45) were not different between the NOSE and CL surgery groups.

**Conclusions:** Compared with CL surgery, NOSE may be a safe procedure, and can achieve similar oncological outcomes. Large multicenter RCTs are needed to provide high-level, evidence-based results in NOSE-treated patients and to determine the risk of local recurrence.

## Introduction

Laparoscopic technology has been widely used to treat colorectal cancer (CRC) over the past two decades, and many studies have demonstrated the advantages of laparoscopic surgery and have suggested that it is a less traumatic procedure, with similar oncological outcomes to those of open surgery (1–3). However, current laparoscopic colectomy is considered to be laparoscopically assisted surgery and not a totally intra-abdominal procedure, as it inevitably extends the incision by about 5–8 cm for specimen extraction and intestinal anastomosis (4, 5). Moreover, the laparotomy incision is also a source of post-operative morbidity, such as pain, wound infection, and incisional hernia (6–8).

In an attempt to further reduce surgical trauma, minimally invasive surgery has undergone unprecedented development. Laparoscopic natural orifice specimen extraction (NOSE) surgery is widely regarded as one of the representative new technologies in minimally invasive surgery (4, 9–12). It combines the concept of incisionless surgery and laparoscopy to complete intra-abdominal procedures (including exploration, dissection, and resection of lesions) and uses a natural orifice as a delivery route for specimen extraction without laparotomy incision (13). Compared with other minimally invasive techniques, laparoscopic colorectal surgery with NOSE adopts a transabdominal approach, which is more in line with the surgeon's practice and is easier to operate (5, 14–16). Recently, several studies have reported that laparoscopic NOSE surgery results in significantly fewer perioperative complications and faster recovery of gastrointestinal function (4, 12). However, the safety and oncological outcomes of laparoscopic colorectal surgery with NOSE are unclear. Therefore, we conducted a meta-analysis to determine the safety and oncological outcomes of laparoscopic NOSE surgery compared with conventional laparoscopic (CL) surgery for colorectal diseases.

## Methods

### Search Strategy

Two independent researchers systematically searched studies in PubMed, EMBASE, and Cochrane databases from January 1990 to September 1, 2018. The search keywords used were “colorectal diseases,” “laparoscopic surgery,” “natural orifice specimen extraction,” “transvaginal specimen extraction,” “transanal specimen extraction,” and “transrectal specimen extraction.” According to the different requirements of each database, the search strategy was correspondingly changed. Potentially relevant articles were also screened from the references of relevant studies.

### Inclusion and Exclusion Criteria

Studies were included if they conformed to the principle of PICO (S) [participants, interventions, comparisons, outcomes, (study design)] (17). Inclusion criteria were as follows: (1) participants: patients were diagnosed with colorectal diseases, either benign or malignant; (2) interventions: totally intra-abdominal laparoscopic colorectal surgery, with the specimen extracted via the rectum, vagina or anus; (3) comparisons: laparoscopic colorectal surgery, with the specimen extracted through the abdominal wall; (4) outcomes: 5-year disease-free survival (DFS), lymph node harvest, proximal margin, distal margin, operation time, hospital stay, total perioperative complications, pain score, time to first flatus, anastomotic leakage, surgical site infection (SSI), blood loss, and intra-abdominal abscess; (5) study design: randomized controlled trials (RCTs), prospective non-randomized trials and retrospective trials based on NOSE. Exclusion criteria were as follows: (1) traditional open surgery; (2) non-colorectal diseases; (3) transanal total mesorectal excision surgery; (4) lack of data, or inability to obtain original data from the author; (5) case reports, letters, reviews, conference abstracts, animal experiments, and expert opinions; (6) studies not written in English or Chinese were excluded.

### Data Extraction and Quality Assessment

Two researchers independently extracted data from the studies. For each included study, the following information was extracted: first author, year of publication, country of origin, number of patients, characteristics of the patients [age, gender, body mass index [BMI], clinical TNM stage, diseases type, extent of resection, specimen extraction approach, location of diseases], study type, information on outcome (primary outcomes: 5-year DFS, lymph node harvest, anastomotic leakage, intra-abdominal abscess, and SSI; secondary outcomes: operation time, hospital stay, pain score, time to first flatus, total perioperative complications, blood loss, proximal margin, and distal margin). If there were any doubts or disagreements regarding outcomes, these studies were submitted to a third researcher for arbitration. For retrospective and prospective non-randomized studies, the Newcastle–Ottawa Scale (NOS) was used (18). The assessment of bias in the RCTs was based on the Cochrane Risk of Bias tool (19).

### Statistical Analysis

Review Manager Version 5.3 software (Cochrane Collaboration, Oxford, UK) was used for statistical analysis. For continuous variables, weighted mean difference (WMD) was used. Odds ratio (OR) was used to express dichotomous variables. The hazard ratio (HR) of 5-year DFS was calculated from survival curves using the methods presented by Tierney et al. (20). The confidence interval (CI) was set at 95%, and *P* < 0.05 was considered statistically significant. The Chi-square test or Cochrane Q test was used to calculate heterogeneity, and *I*^2^ <50% and *P* > 0.10 were defined as non-significant heterogeneity, and such data were evaluated using the fixed effect model; otherwise, the random effect model was used (21, 22). Subgroup analyses were conducted according to the different study types [RCT and NRCT (non-randomized controlled trial)]. A sensitivity analysis was carried out to evaluate the stability of the outcomes. In addition, publication bias was assessed by Begg's funnel plots and Begg's test (STATA, version 12.0) (23).

## Results

### Study Selection and Characteristics

**Figure 1:**
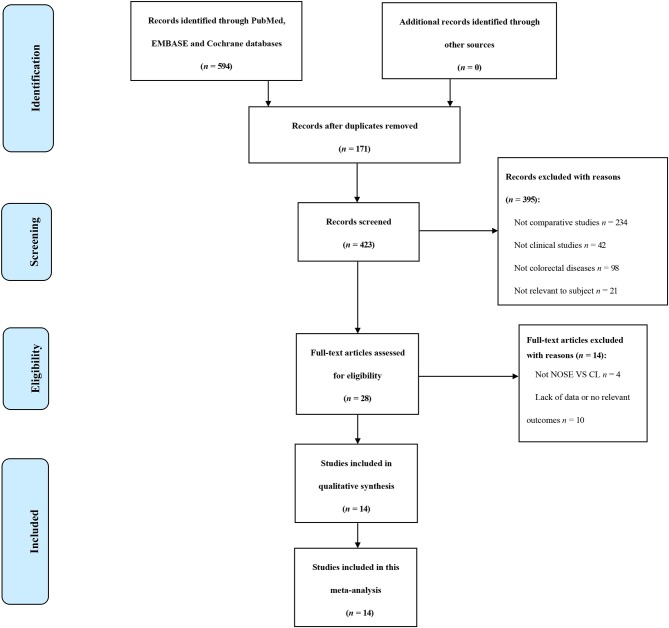
Flow diagram of study selection.

**Figure 2:**
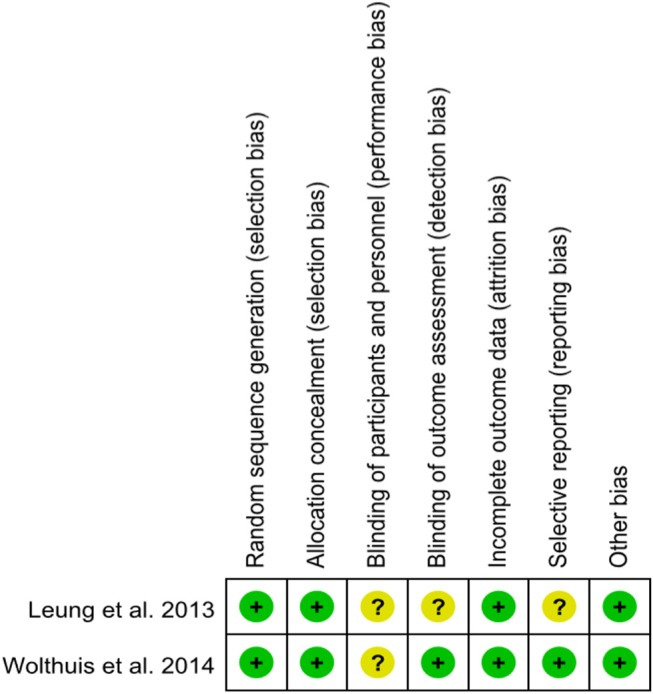
Risk of bias of randomized controlled trials assessed with the Cochrane Risk of Bias tool.

**Figure 3:**
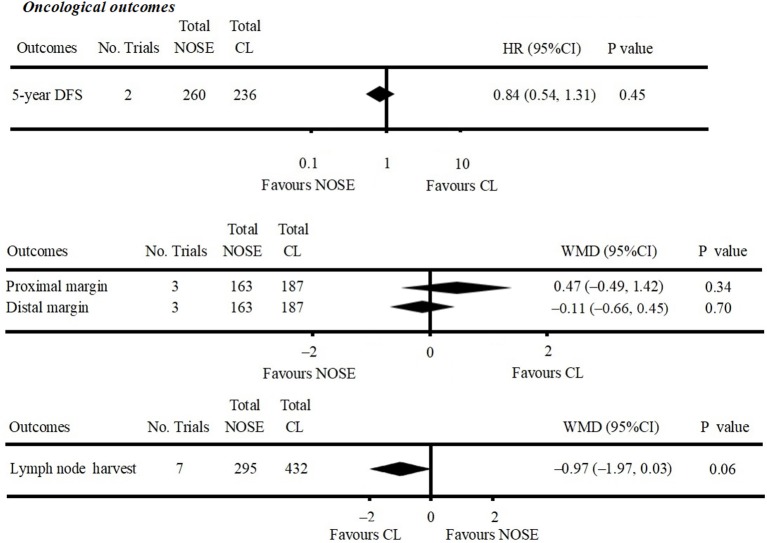
Forest plot of oncological outcomes following natural orifice specimen extraction (NOSE) compared with conventional laparoscopic (CL) surgery.

**Figure 4:**
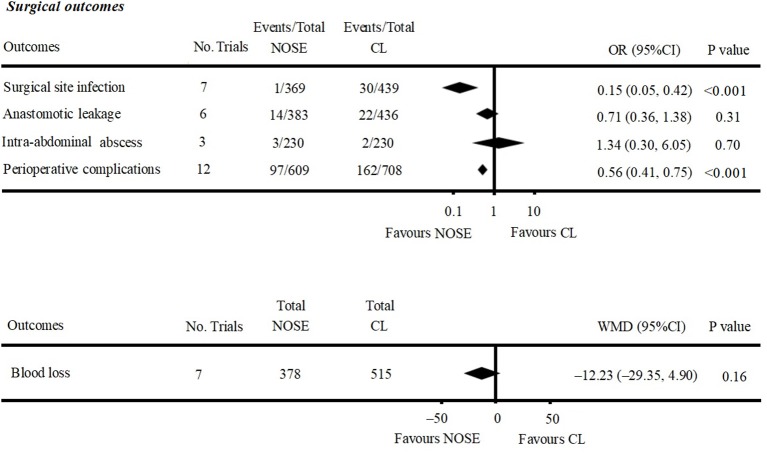
Forest plot of surgical outcomes following NOSE compared with CL.

**Figure 5:**
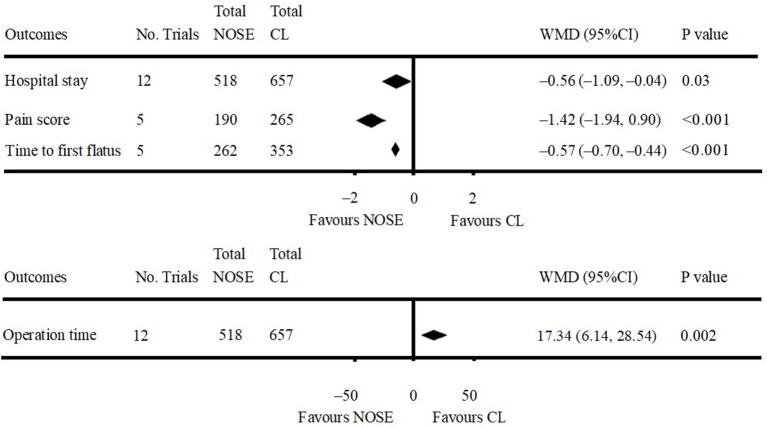
Forest plot of other outcomes following NOSE compared with CL.

A total of 14 studies were included in the meta-analysis [two RCTs (12, 24), seven retrospective studies (4, 5, 10, 11, 25–27), and five prospective non-randomized studies (9, 16, 28–30)]. A flow diagram of study selection is shown in Figure 1. Nine studies were from Asia, four from Europe and one from North America. A total of 1,435 patients were included; 660 in the NOSE group and 775 in the CL surgery group. The two groups were similar in terms of age (*P* = 0.12), body mass index (BMI, *P* = 0.15), and extent of resection (*P* = 0.86). In the included studies, the main steps of NOSE and CL regarding exploration, mobilization, and dissection were the same. NOSE involves a natural orifice for specimen extraction. However, CL surgery involves specimen extraction through the abdominal wall. The basic characteristics of the studies included are summarized in Table 1. All non-randomized studies had a NOS score of ≥5, and RCTs had a low risk of bias. Quality assessment results of the included studies are shown in Table 2, Figure 2, respectively. Forest plots of all the outcomes are shown in Figures 3–**5** and Supplementary Figures 1–10. The meta-analysis of the main endpoints is summarized in Table 3. The meta-analysis of endpoints for cancers is shown in Table 4, Supplementary Figures 1A, 4A–I.

**Table 1:** Basic characteristics of the included studies.

**References**	**Year**	**Country/area**	**Patients (NOSE/CL)**	**Gender (NOSE/CL) (M/F)**		**Age ^a^(NOSE/CL)**		**BMI ^a^ (NOSE/CL)**		**Study type**	**Approach**	**Disease type**	**cTNM**	**Extent of resection (NOSE/CL)**	**Location**
Hisada et al. (4)	2014	Japan	20/50	12/8	NA	63.7 ± 9.0	66.3 ± 11.0	NA	NA	RT	TS	Mal	I-III	NA	Rec
Park et al. (5)	2017	Korea	138/138	32/106	41/97	60.3 ± 10.6	60.4 ± 11.3	23.4 ± 2.9	23.3 ± 3.2	RT	TS /TV	Mal	0-III	NA	Rec
Costantino et al. (9)	2011	France	17/9	6/11	4/5	60.1 ± 9.4	59.5 ± 12.6	25.5 ± 3.0	30.5 ± 4.2	PNT	TS	Ben	NA	NA	SC
Award et al. (10)	2014	USA	20/20	20 (F)	20 (F)	63.6 ± 9.0	66.9 ± 8.9	25.1 ± 6.7	31.6 ± 8.3	RT	TV	Ben/Mal	I-III	24.4 ± 5.9/40.1 ± 26.8	RC
Zhang et al. (11)	2014	China	65/132	32/33	57/75	56.1 ± 9.3	55.5 ± 9.5	23.7 ± 2.9	23.1 ± 3.1	RT	TS	Mal	I-III	NA	SC, Rec
Leung et al. (12)	2013	China	35/35	13/22	12/23	62 (51–86)	72 (49–84)	NA	NA	RCT	TS	Mal	NA	NA	LC
Park et al. (16)	2011	Korea	34/34	34 (F)	34(F)	61.0 ± 11.2	63.6 ± 11.6	23.9 ± 3.1	23.1 ± 2.7	PNT	TV	Mal	I-III	NA	RC
Wolthuis et al. (24)	2014	Belgium	20/20	5/15	10/10	54 (31–72)	58 (40–73)	23.5 (18–29)	24 (20–29)	RCT	TR	Ben/Mal	NA	25 (12–44)/18 (12–33)	SC
Denost et al. (25)	2015	France	122/98	70/52	69/29	63 (20–90)	65 (25–85)	24.3 (17.3–33.6)	25.8 (18.8–38.3)	RT	TS	Mal	0-III	NA	Rec
Saurabh et al. (26)	2017	Taiwan	82/106	47/35	65/41	63.3 ± 13.9	64.7 ± 10.9	24.4 ± 4.2	24.4 ± 3.2	RT	TS	Ben/Mal	I-III	16 ± 5.3/15.3 ± 5.3	SC, Rec
Xu et al. (27)	2016	China	23/23	13/10	13/10	63.0 ± 9.4	63.5 ± 13.5	22.2 ± 2.7	22.2 ± 3.3	RT	TS	Mal	0-III	NA	LC, Rec
Christoforidis et al. (28)	2012	Switzerland	10/20	3/7	10/10	47 (26–62)	56 (38–81)	27.6 (19.7–30.9)	26.4 (19.4–31.6)	PNT	TR	Ben	NA	NA	LC, Rec
Kim et al. (29)	2014	Korea	58/58	58 (F)	58 (F)	62.8 ± 9.0	63.2 ± 10.7	23.5 ± 2.9	23.2 ± 3.3	PNT	TV	Mal	I-III	NA	LC
Xing et al. (30)	2017	China	16/32	12/4	24/8	61.9 ± 11.8	62.4 ± 12.0	23.1 ± 1.2	23.9 ± 1.7	PNT	TS	Mal	I-III	18.2 ± 4.8/19.8 ± 5.7	SC

NOSE, natural orifice specimen extraction; CL, conventional laparoscopic surgery;

a *, mean ± SD or median (range); SD, standard deviation; BMI, body mass index; NA, not available; RCT, randomized controlled trial; RT, retrospective trial; PNT, prospective non-randomized trial; cTNM, clinical TNM stage; M, male; F, female; Ben, benign; Mal, malignant; SC, sigmoid colon; LC, left colon; RC, right colon; Rec, rectum; TS, transanal; TV, transvaginal; TR, transrectal; ref, reference*.

**Table 2:** Newcastle-Ottawa Scale for bias risk assessment of retrospective and prospective non-randomized studies.

**References**	**Case definition**	**Representativeness**	**Control selection**	**Definition of controls**	**Comparability**	**Ascertainment of exposure**	**SMACC**	**Non-response rate**	**Total**
Hisada et al. (4)	1	1	1	1	1	1	1	0	7
Park et al. (5)	1	1	1	0	2	1	1	1	8
Costantino et al. (9)	1	1	1	1	1	1	1	0	7
Award et al. (10)	1	1	1	1	1	1	1	0	7
Zhang et al. (11)	1	1	1	0	1	1	1	0	6
Park et al. (16)	1	1	1	0	1	1	1	0	6
Denost et al. (25)	1	1	1	0	2	1	1	1	8
Saurabh et al. (26)	1	1	1	0	1	1	1	0	6
Xu et al. (27)	1	0	1	0	1	1	1	0	5
Christoforidis et al. (28)	1	1	1	1	1	1	1	0	7
Kim et al. (29)	1	1	1	0	1	1	1	1	7
Xing et al. (30)	1	0	1	0	1	1	1	0	5

*SMACC, same method of ascertainment for cases and controls*.

**Table 3:** Meta-analysis of main endpoints.

**Endpoints**	**No. of patients**	**No. of trials (No. of RCTs)**	**NOSE**	**CL**	**HR/OR/WMD (95%CI)**	***P*-value**	***I^**2**^* (%)**	***P-*value for heterogeneity**
**ONCOLOGICAL OUTCOMES (REFERENCES)**								
Five-year DFS (5, 25)	496	2 (0)	260	236	0.84 (0.54 to 1.31)	0.45	0	0.83
Lymph node harvest (4, 10, 11, 16, 26, 29, 30)	727	7 (0)	295	432	−0.97 (−1.97 to 0.03)	0.06	0	0.76
Proximal margin, cm (26, 27, 29)	350	3 (0)	163	187	0.47 (−0.49 to 1.42)	0.34	0	0.72
Distal margin, cm (26, 27, 29)	350	3 (0)	163	187	−0.11 (−0.66 to 0.45)	0.70	24	0.27
**SAFETY OUTCOMES**								
Surgical site infection (4, 5, 10, 12, 26, 29, 30)	808	7 (1)	369	439	0.15 (0.05 to 0.42)	<0.001	0	0.99
Anastomotic leakage (5, 9, 11, 26, 27, 29)	819	6 (0)	383	436	0.71 (0.36 to 1.38)	0.31	0	0.96
Blood loss, ml (4, 5, 11, 16, 26, 27, 30)	893	7 (0)	378	515	−12.23 (−29.35 to 4.90)	0.16	89	<0.001
Intra-abdominal abscess (5, 16, 29)	460	3 (0)	230	230	1.34 (0.30 to 6.05)	0.70	0	0.47
Total perioperative complications (4, 5, 9–11, 16, 24–29)	1,317	12 (1)	609	708	0.56 (0.41 to 0.75)	<0.001	15	0.30
**OTHER OUTCOMES**								
Operation time, min (4, 5, 9–12, 16, 26–30)	1,175	12 (1)	518	657	17.34 (6.14 to 28.54)	0.002	81	<0.001
Hospital stay, day (4, 5, 9–12, 16, 26–30)	1,175	12 (1)	518	657	−0.56 (−1.09 to −0.04)	0.03	54	0.01
Pain score (9, 11, 16, 29, 30)	455	5 (0)	190	265	−1.42 (−1.94 to 0.90)	<0.001	85	<0.001
Time to first flatus, day (11, 16, 26, 27, 29)	615	5 (0)	262	353	−0.57 (−0.70 to −0.44)	<0.001	0	0.48

*NOSE, natural orifice specimen extraction; CL, conventional laparoscopic surgery; HR, hazard ratio; OR, odds ratio; WMD, weighted mean difference; 95%CI; 95% confidence interval; DFS, diseases-free survival*.

**Table 4:** Meta-analysis of endpoints for cancers.

**Endpoints (references)**	**No. of patients**	**No. of trials (No. of RCTs)**	**NOSE**	**CL**	**HR/OR/WMD (95%CI)**	***P*-value**	***I^**2**^* (%)**	***P*-value for heterogeneity**
Five-year DFS (5, 25)	496	2 (0)	260	236	0.84 (0.54 to 1.31)	0.45	0	0.83
Lymph node harvest (4, 11, 16, 29, 30)	499	5 (0)	193	306	−1.15 (−2.40 to 0.11)	0.07	0	0.75
Proximal margin, cm (27, 29)	162	2 (0)	81	81	1.24 (−1.03 to 3.50)	0.28	0	0.74
Distal margin, cm (27, 29)	162	2 (0)	81	81	−0.48 (−1.21 to 0.25)	0.20	0	0.59
Surgical site infection (4, 5, 12, 29, 30)	580	5 (1)	267	313	0.14 (0.04 to 0.46)	0.001	0	0.98
Anastomotic leakage (5, 11, 27, 29)	605	4 (0)	284	321	0.65 (0.31 to 1.37)	0.25	0	0.88
Blood loss, ml (4, 5, 11, 16, 27, 30)	705	6 (0)	296	409	−12.35 (−32.62 to 7.93)	0.23	91	<0.001
Total perioperative complications (4, 5, 11, 16, 26, 27, 29)	993	7 (0)	460	533	0.52 (0.37 to 0.73)	<0.001	0	0.46
Operation time, min (4, 5, 11, 12, 16, 27, 29, 30)	891	8 (1)	389	502	13.70 (0.97 to 26.43)	0.03	83	<0.001
Hospital stay, day (4, 5, 11, 12, 16, 27, 29, 30)	891	8 (1)	389	502	−0.64 (−1.19 to −0.09)	0.02	55	0.03

*NOSE, natural orifice specimen extraction; CL, conventional laparoscopic surgery; OR, odds ratio; WMD, weighted mean difference; 95%CI; 95% confidence interval; Unite; min, minute*.

### Oncological Outcomes

Five-year DFS was reported in two studies (5, 25). Both of which were NRCTs. There was no significant difference in 5-year DFS between the NOSE and CL surgery groups (HR = 0.84; 95% CI 0.54 to 1.31; *P* = 0.45). No significant heterogeneity was observed (*I*^2^ = 0%); therefore, the fixed effect model was used (Figure 3, Supplementary Figure 1A).

A total of seven studies reported lymph node harvest (4, 10, 11, 16, 26, 29, 30). All of which were NRCTs. There was no significant difference in lymph node harvest between the two groups (WMD = −0.97; 95% CI −1.97 to 0.03; *P* = 0.06). No significant heterogeneity was observed (*I*^2^ = 0%); therefore, the fixed effect model was used (Figure 3, Supplementary Figure 1B).

Data on proximal and distal margins were available in three studies (26, 27, 29). All of which were NRCTs. There was no significant difference in proximal margin (WMD = 0.47; 95% CI −0.49 to 1.42; *P* = 0.34) (Figure 3, Supplementary Figure 1C) and distal margin (WMD = −0.11; 95% CI −0.66 to 0.45; *P* = 0.70) (Figure 3, Supplementary Figure 1D). No significant heterogeneity in proximal margin (*I*^2^ = 0%) and distal margin (*I*^2^ = 24%) was observed; therefore, the fixed effect model was used.

### Safety Outcomes

A total of seven studies reported SSIs (4, 5, 10, 12, 26, 29, 30). There were fewer SSIs in the NOSE group, and the difference was significant (OR = 0.15; 95% CI 0.05 to 0.42; *P* < 0.001). No significant heterogeneity was observed (*I*^2^ = 0%); therefore, the fixed effect model was used. The pooled OR for the NRCT subgroup was 0.16 (95% CI 0.05 to 0.48; *P* = 0.001, *I*^2^ = 0%) (Figure 4, Supplementary Figure 2A).

Anastomotic leakage was reported in six studies (5, 9, 11, 26, 27, 29). All of which were NRCTs. There was no significant difference in anastomotic leakage between the NOSE and CL surgery groups (OR = 0.71; 95% CI 0.36 to 1.38; *P* = 0.31). No significant heterogeneity was observed (*I*^2^ = 0%); therefore, the fixed effect model was used (Figure 4, Supplementary Figure 2B).

A total of seven studies reported blood loss (4, 5, 11, 16, 26, 27, 30). All of which were NRCTs. There was no significant difference between the two groups (WMD = −12.23; 95% CI −29.35 to 4.90; *P* = 0.16). Heterogeneity was observed (*P* for heterogeneity <0.001, *I*^2^ = 89%); therefore, the random effect model was used (Figure 4, Supplementary Figure 2C).

Data on intra-abdominal abscess were included in three studies (5, 16, 29). All of which were NRCTs. There was no significant difference between the two groups (OR = 1.34; 95% CI 0.30–6.05; *P* = 0.70). No significant heterogeneity was observed (*I*^2^ = 0%); therefore, the fixed effect model was used (Figure 4, Supplementary Figure 2D).

Data on total perioperative complications (such as wound infection, anastomotic leakage, ischemia, bleeding, ileus, and anal dysfunction) were reported in 12 studies (4, 5, 9–11, 16, 24–29). The results showed that the NOSE group had fewer complications than the CL surgery group (OR = 0.56; 95% CI 0.41 to 0.75; *P* < 0.001). The difference was significant. No obvious heterogeneity was observed (*I*^2^ = 15%); therefore, the fixed effect model was used. The pooled OR for the NRCT subgroup was 0.55 (95% CI 0.41–0.74; *P* < 0.001, *I*^2^ = 21%) (Figure 4, Supplementary Figure 2E).

### Other Outcomes

Data on operation time was available in 12 studies (4, 5, 9–12, 16, 26–30). Compared to the NOSE group, the operation time was shorter in the CL surgery group (WMD = 17.34; 95% CI 6.14–28.54; *P* = 0.002). Significant heterogeneity was observed (*P* for heterogeneity <0.001, *I*^2^ = 81%); therefore, the random effect model was used. The pooled WMD for the NRCT subgroup was 18.83 (95% CI 6.48–31.17; *P* = 0.003, *I*^2^ = 82%) (Figure 5, Supplementary Figure 3A).

A total of 12 studies reported data on hospital stay (4, 5, 9–12, 16, 26–30). Patients in the NOSE group had a reduced hospital stay compared with patients in the CL surgery group. The difference was significant (WMD = −0.56; 95% CI −1.09 to −0.04; *P* = 0.03). Significant heterogeneity was observed (*P* for heterogeneity = 0.01, *I*^2^ = 54%); therefore, the random effect model was used. The pooled WMD for the NRCT subgroup was −0.66 (95% CI −1.22 to −0.10; *P* = 0.02, *I*^2^ = 50%) (Figure 5, Supplementary Figure 3B).

Five studies reported pain score using the visual analog scale (VAS) on post-operative day 1 (9, 11, 16, 29, 30). All of which were NRCTs. The NOSE group had a lower VAS score than the CL surgery group. The difference was significant (WMD = −1.42; 95% CI −1.94 to −0.90; *P* < 0.001). Significant heterogeneity was observed (*P* for heterogeneity <0.001, *I*^2^ = 85%); therefore, the random effect model was used (Figure 5, Supplementary Figure 3C).

Data on time to first flatus was included in five studies (11, 16, 26, 27, 29). All of which were NRCTs. Compared with the CL surgery group, time to first flatus was shorter in the NOSE group. The difference was significant (WMD = −0.57; 95% CI −0.70 to −0.44; *P* < 0.001). No significant heterogeneity was observed (*I*^2^ = 0%); therefore, the fixed effect model was used (Figure 5, Supplementary Figure 3D).

### Publication Bias

**Figure 6:**
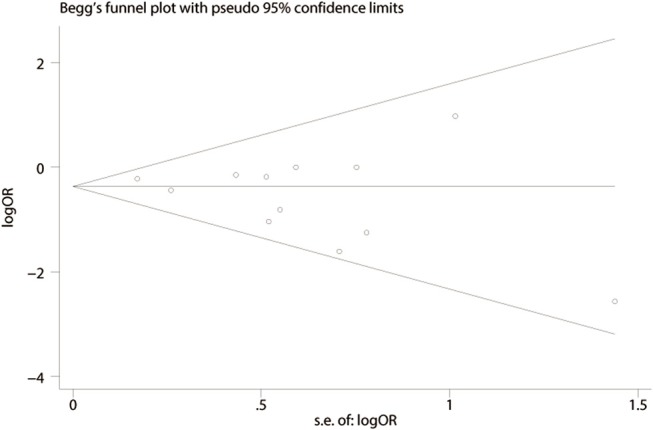
Funnel plot of publication bias based on total perioperative complications (Begg's test, *P* = 0.373).

We performed a funnel plot of the studies included to assess publication bias. No obvious asymmetry was noted and none of the studies were outside the limits of the 95% CI (Figure 6). No significant publication bias among these studies was observed using Begg's test (*P* = 0.373). In addition, a sensitivity analysis was performed using six outcomes (lymph node harvest, SSI, anastomotic leakage, total perioperative complications, operation time, hospital stay) and the results are shown in Table 5. Forest plots based on exclusion criteria in the sensitivity analysis are shown in Supplementary Figures 5–10. Finally, exclusion of any single study and sensitivity analysis based on various exclusion criteria did not affect the pooled results, except hospital stay based on prospective studies.

**Table 5:** Sensitivity analysis of endpoints of interests.

**Endpoints (references)**	**No. of patients**	**No. of trials**	**NOSE**	**CL**	**OR or WMD (95%CI)**	***P*-value**	***I^**2**^* (%)**	***P* value for heterogeneity**
**LYMPH NODE HARVEST**								
All included trails (4, 10, 11, 16, 26, 29, 30)	727	7	295	432	−0.97 (−1.97 to 0.03)	0.06	0	0.76
BMI ≤ 30 (kg/m^2^) (4, 11, 16, 26, 29, 30)	687	6	275	412	−0.87 (−1.89 to 0.16)	0.10	0	0.77
Sample number >30 (11, 16, 26, 29)	569	4	239	330	−0.59 (−1.99 to 0.80)	0.41	0	0.64
Non-RCT (NOS ≥ 6) (4, 10, 11, 16, 26, 29)	679	6	279	400	−0.71 (−1.98 to 0.56)	0.27	0	0.71
Prospective trials (16, 29, 30)	232	3	108	124	−1.11 (−2.59 to 0.38)	0.14	0	0.53
**TOTAL PERIOPERATIVE COMPLICATIONS**								
All included trails (4, 5, 9–11, 16, 24–29)	1,317	12	609	708	0.56 (0.41 to 0.75)	<0.001	15	0.30
BMI ≤ 30 (kg/m^2^) (4, 5, 11, 16, 24, 26, 27, 29)	1,001	8	440	561	0.50 (0.34 to 0.74)	<0.001	0	0.52
Sample number > 30 (5, 11, 16, 25, 26, 29)	1,065	6	499	566	0.57 (0.41 to 0.79)	<0.001	0	0.59
Non-RCT (NOS ≥ 6) (4, 5, 9–11, 16, 25, 26, 28, 29)	1,231	10	566	665	0.57 (0.42 to 0.78)	<0.001	11	0.34
Prospective trials (5, 9, 16, 24, 29)	526	5	267	259	0.58 (0.36 to 0.93)	0.03	0	0.41
**ANASTOMOTIC LEAKAGE**								
All included trails (5, 9, 11, 26, 27, 29)	819	6	383	436	0.71 (0.36 to 1.38)	0.31	0	0.96
BMI ≤ 30 (kg/m^2^) (5, 11, 26, 27, 29)	793	5	366	427	0.68 (0.34 to 1.34)	0.26	0	0.95
Sample number > 30 (5, 11, 26, 29)	747	4	343	404	0.70 (0.35 to 1.42)	0.33	0	0.92
Non-RCT (NOS ≥ 6) (5, 9, 11, 26, 29)	773	5	360	413	0.73 (0.37 to 1.46)	0.38	0	0.94
Prospective trials (5, 29)	392	2	196	196	0.75 (0.31 to 1.78)	0.51	0	0.60
**SURGICAL SITE INFECTION**								
All included trails (4, 5, 10, 12, 26, 29, 30)	808	7	369	439	0.15 (0.05 to 0.42)	<0.001	0	0.99
BMI ≤ 30 (kg/m^2^) (4, 5, 12, 26, 29, 30)	768	6	349	419	0.14 (0.04 to 0.42)	<0.001	0	0.99
Sample number > 30 (5, 12, 26, 29)	650	4	313	337	0.12 (0.03 to 0.45)	0.002	0	1.00
Non-RCT (NOS ≥ 6) (4, 5, 10, 26, 29)	690	5	318	372	0.14 (0.04 to 0.47)	0.001	0	0.99
Prospective trials (5, 12, 29, 30)	510	4	247	263	0.14 (0.04 to 0.53)	0.004	0	0.93
**OPERATION TIME**								
All included trails (4, 5, 9–12, 16, 26–30)	1,175	12	518	657	17.34 (6.14 to 28.54)	0.002	81	<0.001
BMI ≤ 30 (kg/m^2^) (4, 5, 11, 12, 16, 26, 27, 29, 30)	1,079	9	471	608	13.17 (1.86 to 24.47)	0.02	80	<0.001
Sample number > 30 (5, 11, 12, 16, 26, 29)	915	6	412	503	11.08 (0.46 to 21.70)	0.04	72	0.003
Non-RCT (NOS ≥ 6) (4, 5, 9–11, 16, 26, 28, 29)	1,011	9	444	567	14.84 (1.04 to 28.63)	0.03	81	<0.001
Prospective trials (5, 9, 12, 16, 29, 30)	604	6	298	306	17.36 (11.43 to 23.28)	<0.001	0	0.65
**HOSPITAL STAY**								
All included trails (4, 5, 9–12, 16, 26–30)	1,175	12	518	657	−0.56 (−1.09 to −0.04)	0.03	54	0.01
BMI ≤ 30 (kg/m^2^) (4, 5, 11, 12, 16, 26, 27, 29, 30)	1,079	9	471	608	−0.71 (−1.19 to −0.23)	0.004	51	0.04
Sample number > 30 (5, 11, 12, 16, 26, 29)	915	6	412	503	−0.84 (−1.16 to −0.53)	<0.001	43	0.12
Non-RCT (NOS ≥ 6) (4, 5, 9–11, 16, 26, 28, 29)	1,011	9	444	567	−0.88 (−1.21 to −0.56)	<0.001	41	0.09
Prospective trials (5, 9, 12, 16, 29, 30)	604	6	298	306	−0.57 (−1.35 to 0.20)	0.15	62	0.02

*NOSE, natural orifice specimen extraction; CL, conventional laparoscopic surgery; OR, odds ratio; WMD, weighted mean difference; 95%CI; 95% confidence interval; BMI, body mass index*.

## Discussion

This meta-analysis mainly focused on the oncological and safety outcomes of laparoscopic colorectal surgery using NOSE. We found that oncological outcomes and safety outcomes of NOSE were not significantly different to those of CL surgery.

Surgical safety is always an important concern for surgeons. Severe post-operative complications may even lead to failure of the entire operation (31–34). An enterotomy within the peritoneal cavity and insertion of an anvil into the abdominal cavity through a natural orifice are necessary in some approaches of NOSE, which can cause bacteriological concerns (13, 28, 35). Costantino et al. and Wolthuis et al. studied the bacterial positive rate in peritoneal fluid culture and demonstrated that, although NOSE had a higher risk of peritoneal contamination, there were no significant differences in clinical outcomes between the two groups (9, 24). Recently, a multicenter study of 718 cases from China further showed that the incidence of intraperitoneal infection after NOSE was only 0.8% (36). To reduce the risk of peritoneal bacterial contamination, pre-operative administration of prophylactic antibiotics, pre-operative bowel preparation, intraoperative peritoneal irrigation and intraoperative transanal lavage are considered routine procedures in NOSE (15). From our pooled data, SSI was reduced in the NOSE group and the incidence of intra-abdominal abscess was not significantly different between the two groups. Post-operative anastomotic leakage is another severe complication in colorectal surgery, and must be avoided. Several factors are considered to increase the incidence of anastomotic leakage, such as excessive tension in the reconstructed bowel, anastomotic ischemia, and anastomotic technique (4, 33). A circular stapler device and end-to-end anastomosis are commonly used in both groups. However, anastomosis in CL colectomy is performed extracorporeally and differs from that in totally laparoscopic surgery with intracorporeal anastomosis (IA). During laparoscopic left colectomy with extracorporeal anastomosis (EA), exteriorization of the bowel requires greater mobilization of colonic segments and the mesentery, which may result in mesenteric laceration and bleeding, further endangering the blood supply of the anastomotic stoma. However, IA requires less mobilization than EA, and therefore facilitates the achievement of tension-free anastomosis. Recent studies have demonstrated that compared to EA, IA is not associated with a greater incidence of anastomotic leakage (37–39). NOSE has a significant advantage in that it reduces anastomotic leakage in ultra-low rectal anus-preserving surgery (40). Unlike CL surgery in a narrow pelvic cavity, NOSE can evaginate the rectal specimen through the anus to the external, and easily close the distal rectum end under direct vision, further reducing the incidence of anastomotic leakage (41). The incidence of anastomotic leakage across the included studies in the NOSE group was 3.6% and was 5% in the CL group (Figure 4). From the pooled data in the present study, the incidence of anastomotic leakage was not significantly different between the two groups. In summary, we consider that laparoscopic colorectal surgery with NOSE is surgically safe.

Lymph node metastasis, local recurrence (LR) and positive margin are life-threatening conditions in colorectal cancer surgery, often associated with worse overall survival (OS) and DFS (42–47). In this study, we used 5-year DFS to evaluate the long-term oncological safety of the NOSE technique. Anatomically, the distribution of lymphatic vessels is in parallel with the colonic mesenteric vessels. When the pre-resection margin of the bowel is determined, the corresponding mesenteric vessels are ligated, and the adherent lymph nodes are removed accordingly. The exploration, mobilization and dissection steps in NOSE and CL are almost the same, which indicates a similar lymph node harvest in both groups. In our meta-analysis, the number of lymph nodes harvested was not significantly different between the two groups. In addition, the 2017 National Comprehensive Cancer Network guidelines recommend removal of at least 12 lymph nodes during lymphadenectomy for cancer surgery. In all the studies included, more than 12 lymph nodes were removed in each group. Therefore, we suggest that laparoscopic NOSE can achieve adequate lymph node harvest similar to CL surgery. Complying with the principle of tumor-free during surgery is another challenge for NOSE. This concern arises from an incision at the colorectal stump (or vagina) in the abdominopelvic cavity and specimen extraction through a narrow natural orifice which may cause cancer cell implantation, and is a significant issue regarding LR and DFS (48). However, in clinical practice, the following steps are taken to reduce the risk of tumor seeding and peritoneal contamination: distal cytocidal rectal lavage; and a specimen extraction bag or a professional platform [transanal endoscopic operation [TEO] device or transanal endoscopic microsurgery [TEM] device] is inserted during the retrieval phase (15). Moreover, previous studies have confirmed that LR after NOSE is comparable to CL (5, 25). A tumor can achieve distant invasion by intramural spread; therefore, inadequate surgical resection may lead to a positive margin which is an independent factor of DFS (49). Three of the included studies reported surgical margin status (26, 27, 29). All of which showed no positive surgical margin in the NOSE procedures, and the margin was the recommended distance from the center of the tumor (50). From our pooled data, the proximal margin and distal margin in the NOSE group were not significantly different compared to the CL group. We also conclude from this meta-analysis that 5-year DFS in the two treatment groups was not significantly different. Based on the above findings, we suggest that laparoscopic colorectal surgery with NOSE meets the expectations concerning oncological safety.

Previous studies have reported faster gastrointestinal recovery, less post-operative pain and shorter hospital stay following laparoscopic colorectal surgery with NOSE (51–54). The results of our meta-analysis also suggested that the NOSE group had less post-operative pain, shorter hospital stay and shorter time to first flatus. Possible reasons for these advantages are as follows: Laparotomy incision which traumatizes the abdominal wall, is more likely to cause vessel and nerve injury, and lead to increasing post-operative somatic pain (16). Reduction of pain is constructive for post-operative stress which consists of inflammatory cascades, which once activated, may have an adverse influence on recovery and hospital stay (55). Several studies have also reported a decrease in post-operative analgesia requirement, which may be beneficial for faster recovery (24, 26, 29). The NOSE technique is conducted totally intraperitoneal; therefore, avoids intraabdominal organs contacting the external environment, and disturbance in the abdominal cavity is slight (56). In addition, patients in the NOSE group had early ambulation which also led to faster gastrointestinal recovery (11, 57). However, the operation time in the CL surgery group was shorter than that in the NOSE group, probably due to the time needed for purse-string suturing and anastomosis of the colorectal stump (10, 16). One study reported a decreasing trend in operation time, indicating the existence of a learning curve in NOSE (4). Therefore, we are convinced that an experienced surgeon may not need more time to complete this procedure. In conclusion, as an incisionless operation, the NOSE technique can aid early post-operative recovery of gastrointestinal function.

However, there were some limitations in our meta-analysis. Firstly, only two RCTs were included in our study, which may influence the power of pooled results. Secondly, differences in surgical proficiency in NOSE technology, T stage and tumor location may lead to heterogeneity of some results. For instance, operation time ranged from ~105–240 min and hospital stay from about 4.8–12.9 d in the NOSE group. Thirdly, long-term outcomes such as LR and OS are still lacking which could provide further support of oncological safety.

Ma et al. conducted a meta-analysis on NOSE in 2015 (58). The analysis included nine studies and a total of 837 patients, and concluded that laparoscopic colorectal surgery with NOSE can reduce the duration of hospital stay, accelerate post-operative recovery with better cosmetic results, and result in less post-operative pain. However, there are still concerns regarding the surgical and oncological safety of this technique. Therefore, we conducted a meta-analysis of 14 studies including a total of 1,435 patients. Moreover, we analyzed studies only involving malignancies and concluded that the results were consistent with our conclusions. The results of the sensitivity analysis and subgroup analyses also support our conclusions and further provide robust evidence on the reliability of our results. All statistical methods mentioned above add credibility to the pooled results of our meta-analysis. In summary, 5-year DFS, lymph node harvest and surgical margin in the NOSE group were comparable to those in the CL group. Moreover, the NOSE group had similar blood loss and anastomotic leakage to the CL group, and a reduced incidence of SSI and total perioperative complications than the CL group.

In conclusion, laparoscopic colorectal surgery with NOSE can achieve comparable oncological and surgical safety to CL surgery. In addition, the NOSE technique has clear advantages in terms of early recovery of gastrointestinal function. However, large multicenter RCTs are needed in the future to provide high-level, evidence-based results regarding functional outcomes assessing anal or vaginal dysfunction and long-term oncological outcomes to further evaluate the feasibility of the NOSE technique.

## Data Availability

Publicly available datasets were analyzed in this study. These data can be found at the following address: doi. http://doi.org/10.6084/m9.figshare.7856828.

## Author Contributions

R-JL and C-DZ contributed to study design, data extraction, data analysis, and manuscript writing. They also reviewed and revised the paper and approved and submitted the final manuscript. Y-CF reviewed and revised the paper. D-QD wrote, reviewed, and revised the paper and submitted the final manuscript. All authors approved the final manuscript and its submission.

### Conflict of Interest Statement

The authors declare that the research was conducted in the absence of any commercial or financial relationships that could be construed as a potential conflict of interest.
