# Supplementary material for: Safety and Oncological Outcomes of Laparoscopic NOSE Surgery Compared With Conventional Laparoscopic Surgery for Colorectal Diseases: A Meta-Analysis
Source: Front Oncol. 2019 Jul 3;9:597. doi: 10.3389/fonc.2019.00597 (PMC6617713; doi:10.3389/fonc.2019.00597)

**Supplementary Figure legends**

**Supplementary Figure 1A.** Forest plot of 5-year DFS following NOSE compared with CL.

**Supplementary Figure 1B.** Forest plot of lymph node harvest following NOSE compared with CL.

**Supplementary Figure 1C.** Forest plot of proximal margin following NOSE compared with CL.

**Supplementary Figure 1D.** Forest plot of distal margin following NOSE compared with CL.

**Supplementary Figure 2A.** Forest plot of surgical site infection following NOSE compared with CL.

**Supplementary Figure 2B.** Forest plot of anastomotic leakage following NOSE compared with CL.

**Supplementary Figure 2C.** Forest plot of blood loss following NOSE compared with CL.

**Supplementary Figure 2D.** Forest plot of intra-abdominal abscess following NOSE compared with CL.

**Supplementary Figure 2E.** Forest plot of total perioperative complications following NOSE compared with CL.

**Supplementary Figure 3A.** Forest plot of operation time following NOSE compared with CL.

**Supplementary Figure 3B.** Forest plot of hospital stay following NOSE compared with CL.

**Supplementary Figure 3C.** Forest plot of pain score following NOSE compared with CL.

**Supplementary Figure 3D.** Forest plot of time to first flatus following NOSE compared with CL.

**Supplementary Figure 4A.** Forest plot of lymph node harvest based on malignant diseases.

**Supplementary Figure 4B.** Forest plot of proximal margin based on malignant diseases.

**Supplementary Figure 4C.** Forest plot of distal margin based on malignant diseases.

**Supplementary Figure 4D.** Forest plot of surgical site infection based on malignant diseases.

**Supplementary Figure 4E.** Forest plot of anastomotic leakage based on malignant diseases.

**Supplementary Figure 4F.** Forest plot of blood loss based on malignant diseases.

**Supplementary Figure 4G.** Forest plot of total perioperative complications based on malignant diseases.

**Supplementary Figure 4H.** Forest plot of operation time based on malignant diseases.

**Supplementary Figure 4I.** Forest plot of hospital stay based on malignant diseases.

**Supplementary Figure 5A.** Forest plot of lymph node harvest based on sample number > 30.

**Supplementary Figure 5B.** Forest plot of lymph node harvest based on BMI ≤ 30 (kg/m^2^).

**Supplementary Figure 5C.** Forest plot of lymph node harvest based on non-RCTs (NOS ≥ 6).

**Supplementary Figure 5D.** Forest plot of lymph node harvest based on prospective trials.

**Supplementary Figure 6A.** Forest plot of total perioperative complications based on sample number > 30.

**Supplementary Figure 6B.** Forest plot of total perioperative complications based on BMI ≤ 30 (kg/m^2^).

**Supplementary Figure 6C.** Forest plot of total perioperative complications based on non-RCTs (NOS ≥ 6).

**Supplementary Figure 6D.** Forest plot of total perioperative complications based on prospective trials.

**Supplementary Figure 7A.** Forest plot of anastomotic leakage based on sample number > 30.

**Supplementary Figure 7B.** Forest plot of anastomotic leakage based on BMI ≤ 30 (kg/m^2^).

**Supplementary Figure 7C.** Forest plot of anastomotic leakage based on non-RCTs (NOS ≥ 6).

**Supplementary Figure 7D.** Forest plot of anastomotic leakage based on prospective trials.

**Supplementary Figure 8A.** Forest plot of surgical site infection based on sample number > 30.

**Supplementary Figure 8B.** Forest plot of surgical site infection based on BMI ≤ 30 (kg/m^2^).

**Supplementary Figure 8C.** Forest plot of surgical site infection based on non-RCT (NOS ≥ 6).

**Supplementary Figure 8D.** Forest plot of surgical site infection based on prospective trials.

**Supplementary Figure 9A.** Forest plot of operation time based on sample number > 30.

**Supplementary Figure 9B.** Forest plot of operation time based on BMI ≤ 30 (kg/m^2^).

**Supplementary Figure 9C.** Forest plot of operation time based on non-RCTs (NOS ≥ 6).

**Supplementary Figure 9D.** Forest plot of operation time based on prospective trials.

**Supplementary Figure 10A.** Forest plot of hospital stay based on sample number > 30.

**Supplementary Figure 10B.** Forest plot of hospital stay based on BMI ≤ 30 (kg/m^2^).

**Supplementary Figure 10C.** Forest plot of hospital stay based on non-RCTs (NOS ≥ 6).

**Supplementary Figure 10D.** Forest plot of hospital stay based on prospective trials.

**Supplementary Figure 1A**


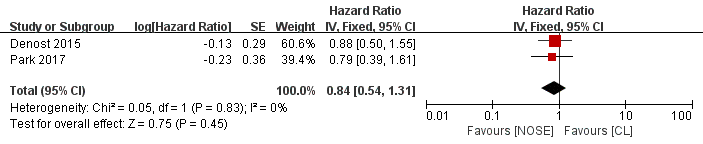


**Supplementary Figure 1B**


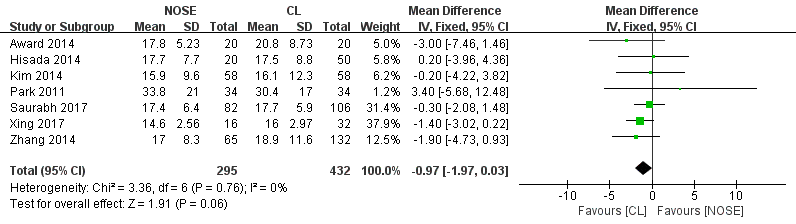


**Supplementary Figure 1C**


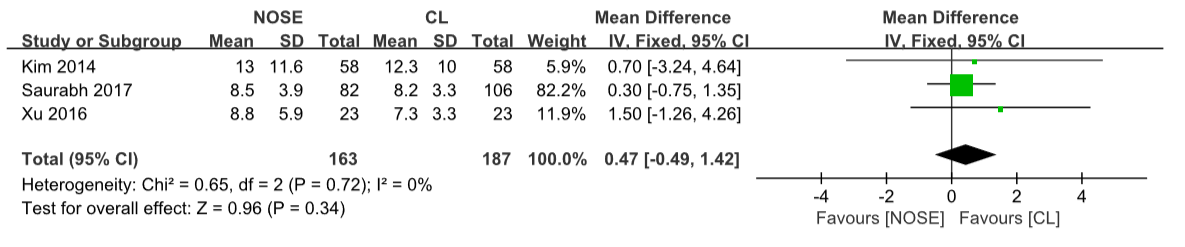


**Supplementary Figure 1D**


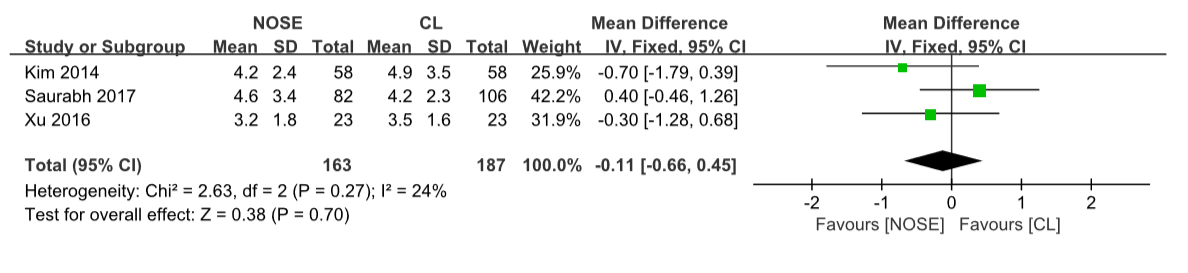


**Supplementary Figure 2A**


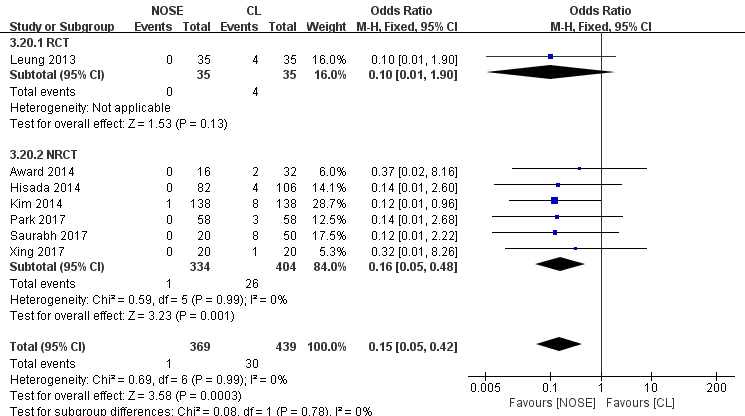


**Supplementary Figure 2B**


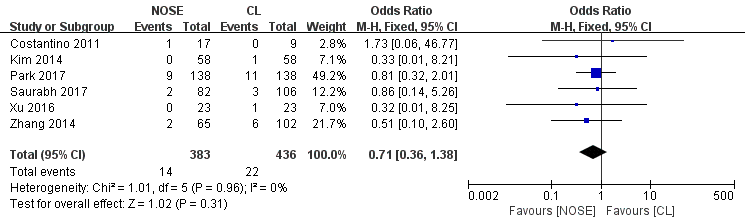


**Supplementary Figure 2C**


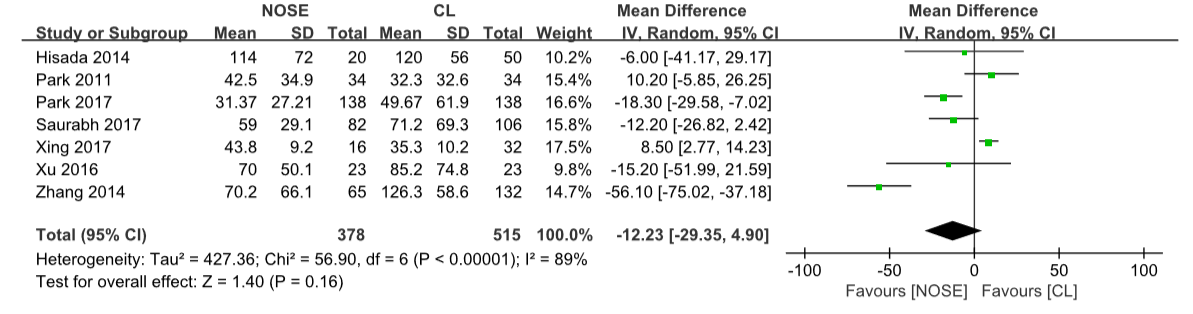


**Supplementary Figure 2D**


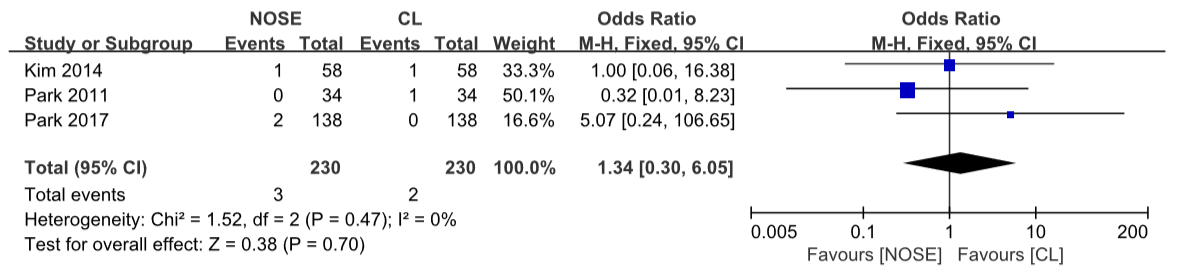


**Supplementary Figure 2E**


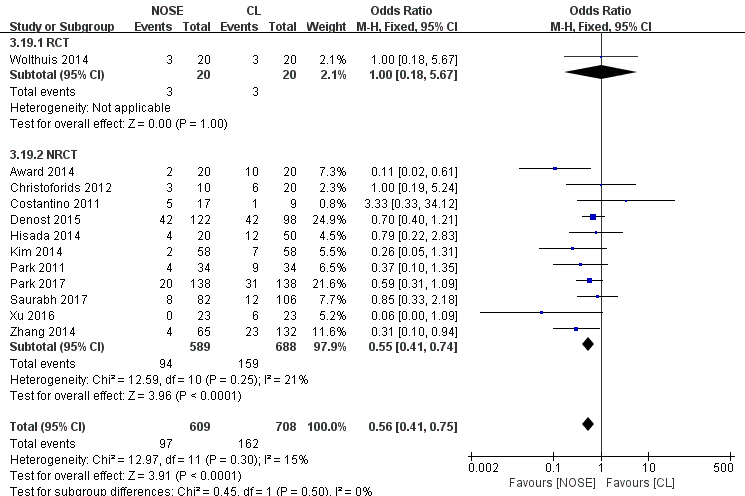


**Supplementary Figure 3A**


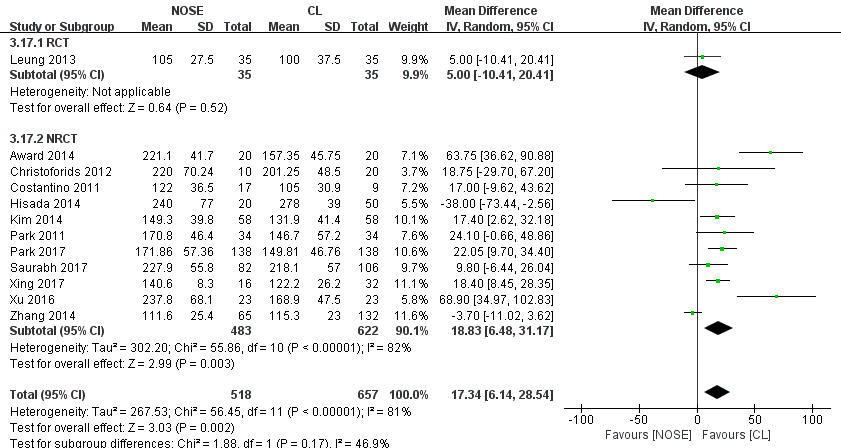


**Supplementary Figure 3B**


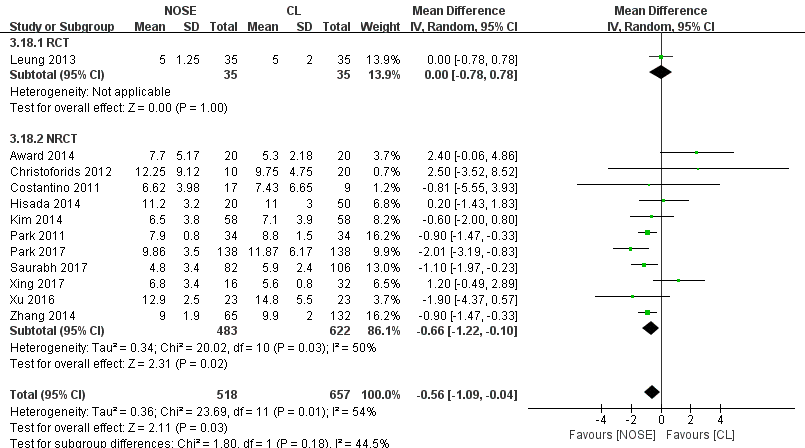


**Supplementary Figure 3C**


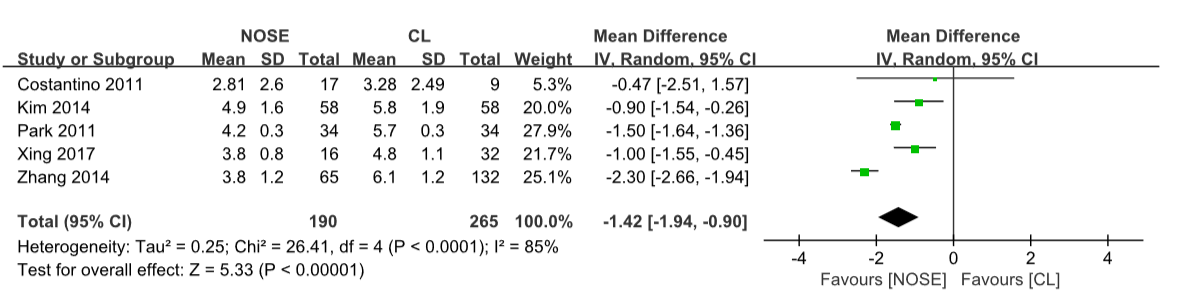


**Supplementary Figure 3D**


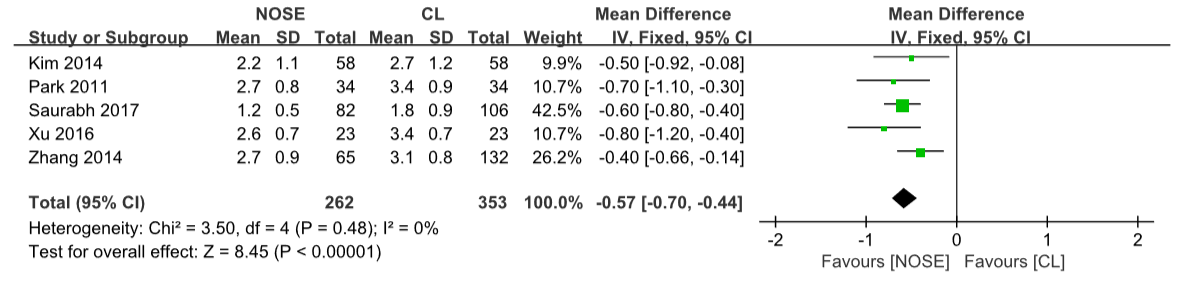


**Supplementary Figure 4A**


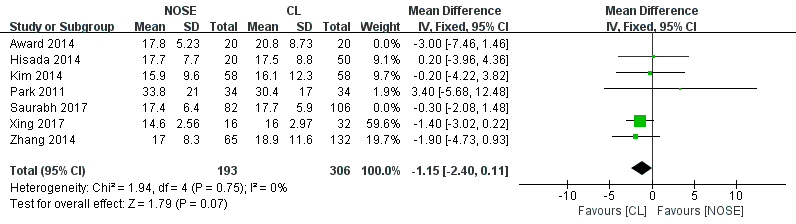


**Supplementary Figure 4B**


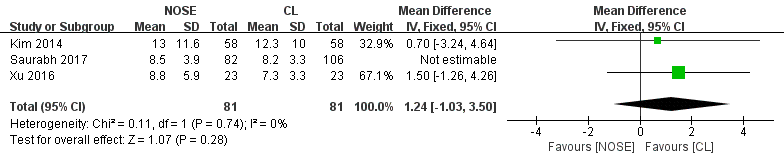


**Supplementary Figure 4C**


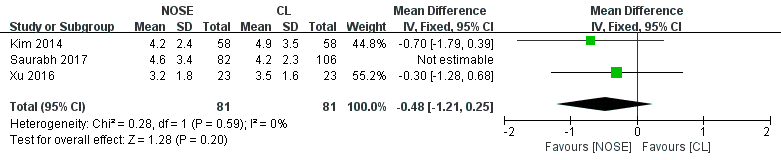


**Supplementary Figure 4D**


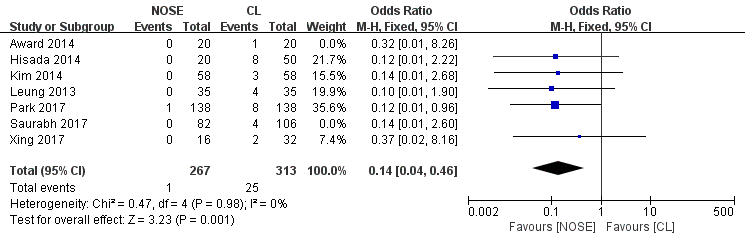


**Supplementary Figure 4E**


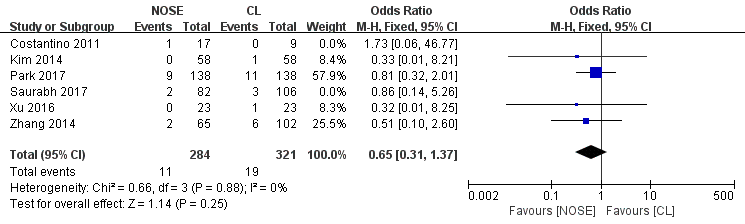


**Supplementary Figure 4F**


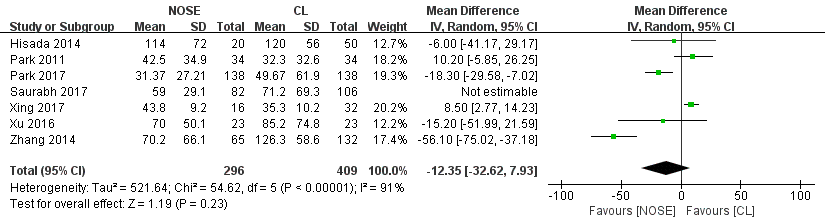


**Supplementary Figure 4G**


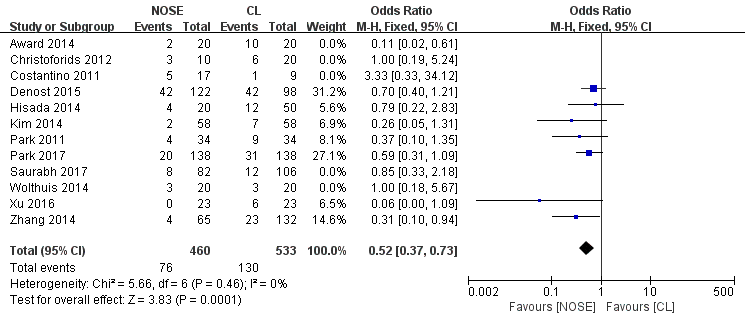


**Supplementary Figure 4H**


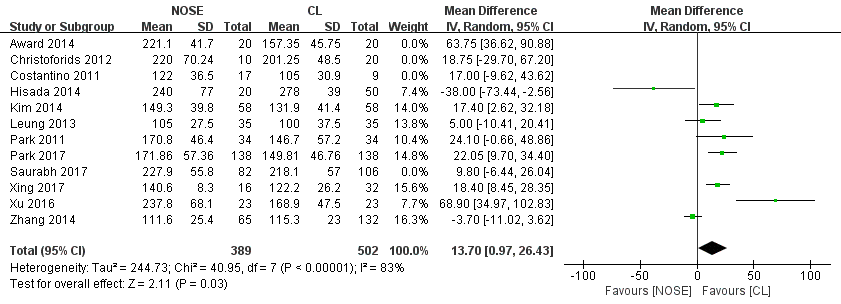


**Supplementary Figure 4I**


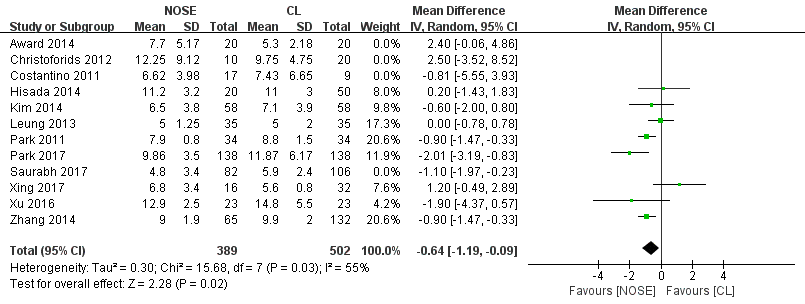


**Supplementary Figure 5A**


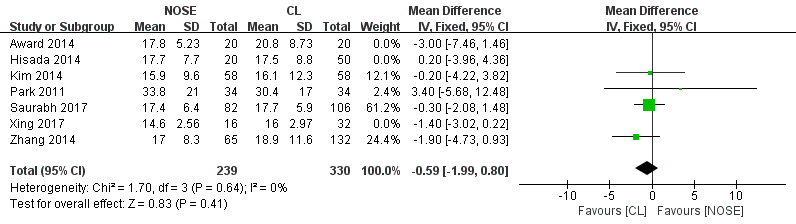


**Supplementary Figure 5B**


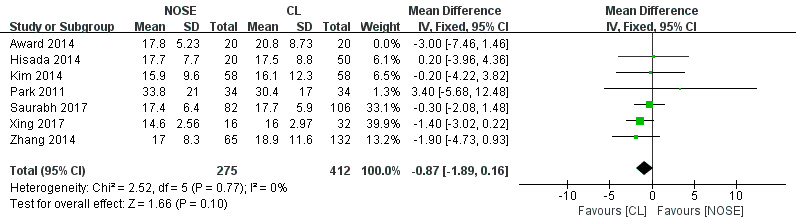


**Supplementary Figure 5C**


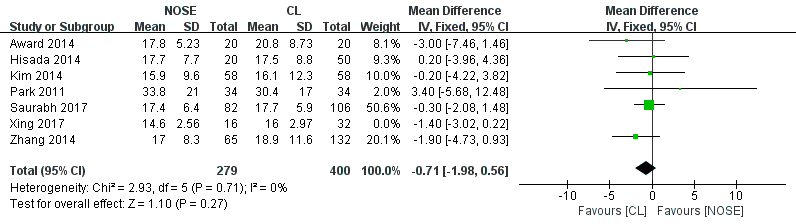


**Supplementary Figure 5D**


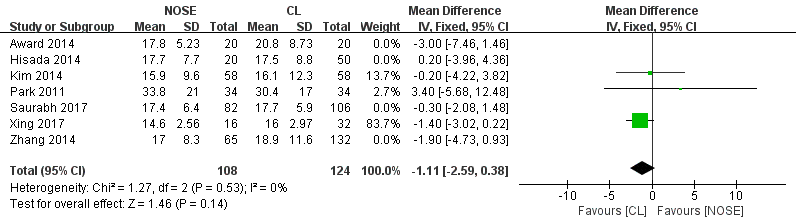


**Supplementary Figure 6A**


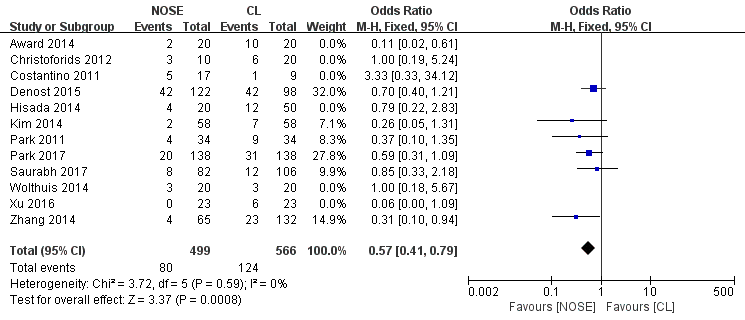


**Supplementary Figure 6B**


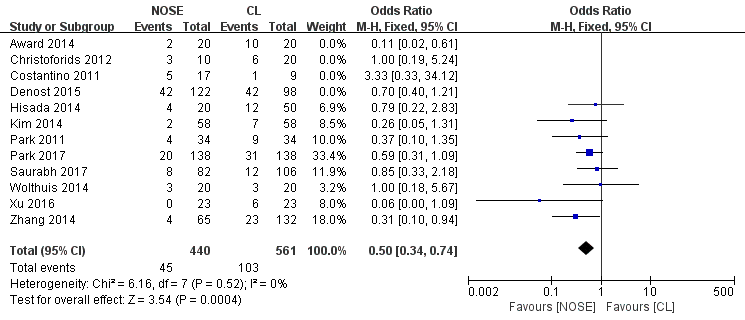


**Supplementary Figure 6C**


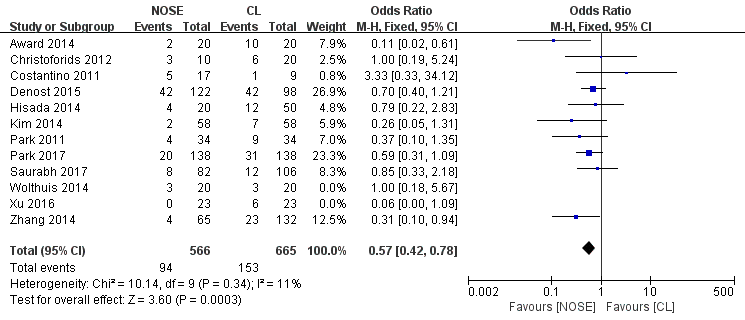


**Supplementary Figure 6D**


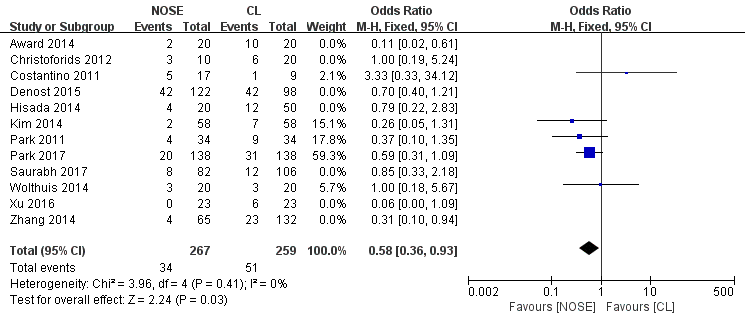


**Supplementary Figure 7A**


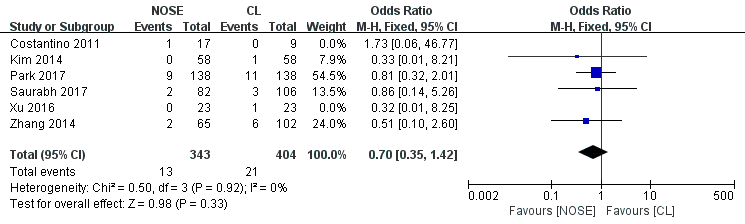


**Supplementary Figure 7B**


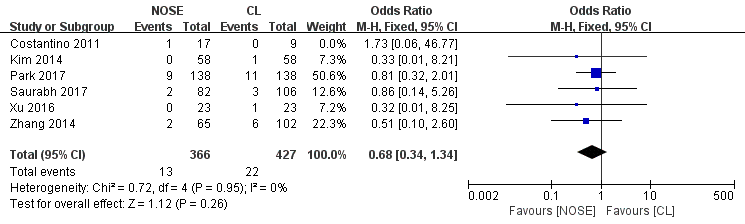


**Supplementary Figure 7C**


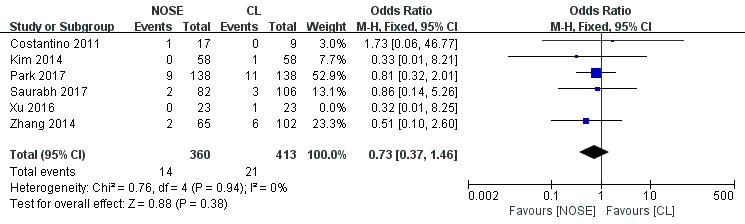


**Supplementary Figure 7D**


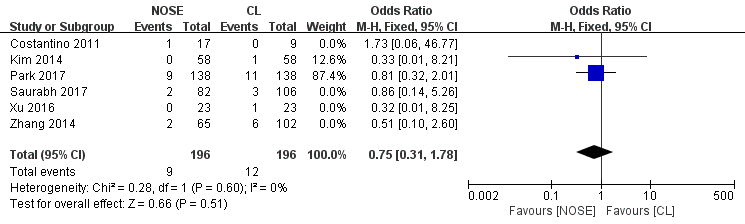


**Supplementary Figure 8A**


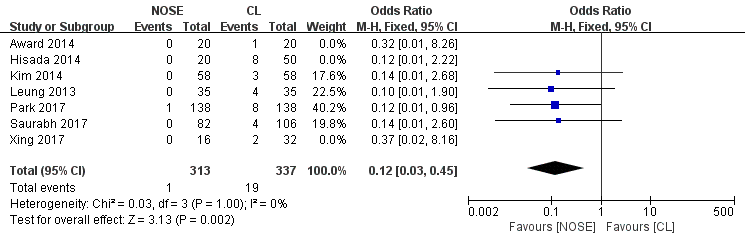


**Supplementary Figure 8B**


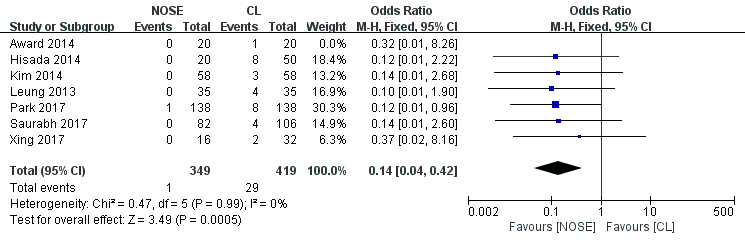


**Supplementary Figure 8C**


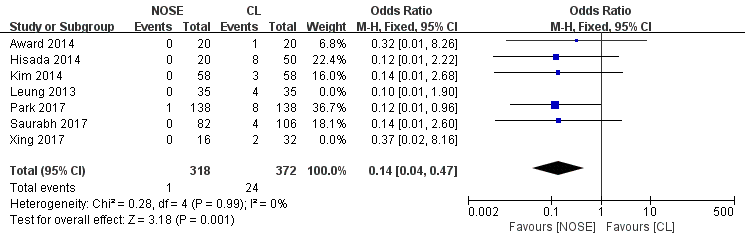


**Supplementary Figure 8D**


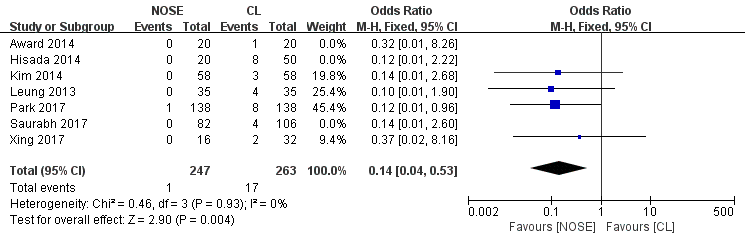


**Supplementary Figure 9A**


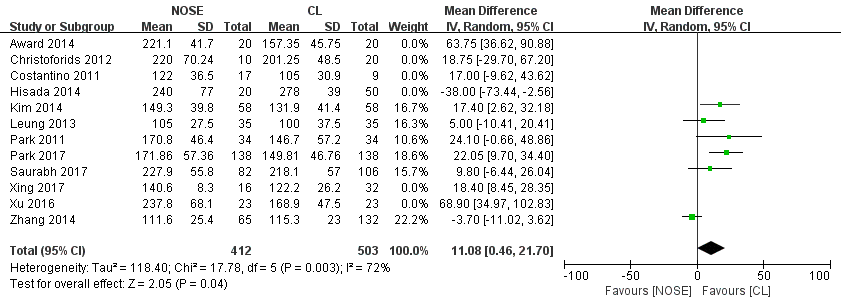


**Supplementary Figure 9B**


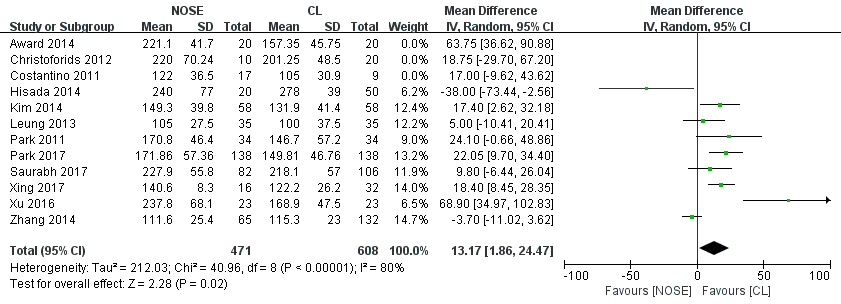


**Supplementary Figure 9C**


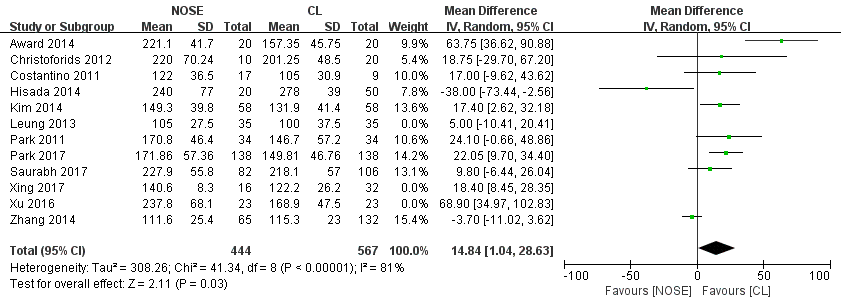


**Supplementary Figure 9D**


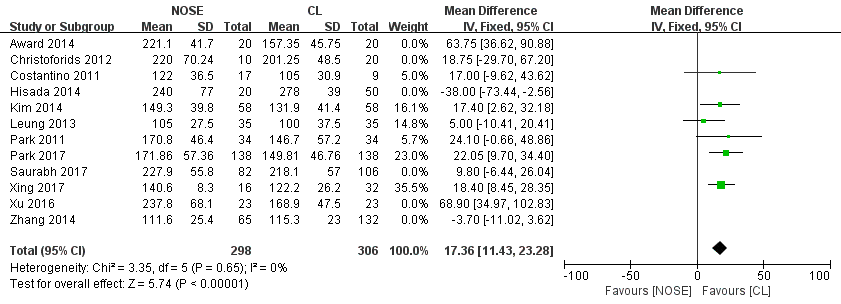


**Supplementary Figure 10A**


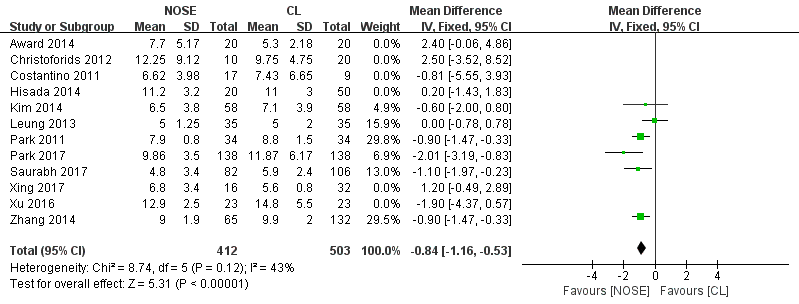


**Supplementary Figure 10B**


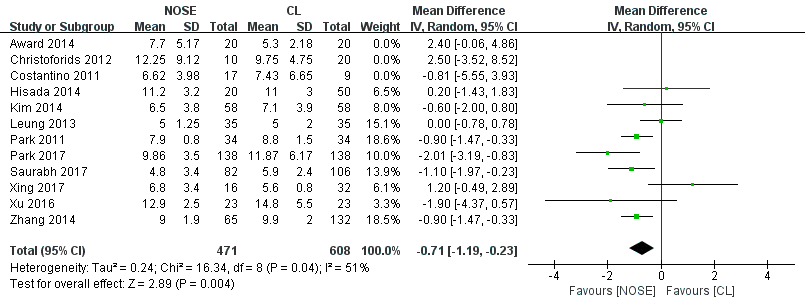


**Supplementary Figure 10C**


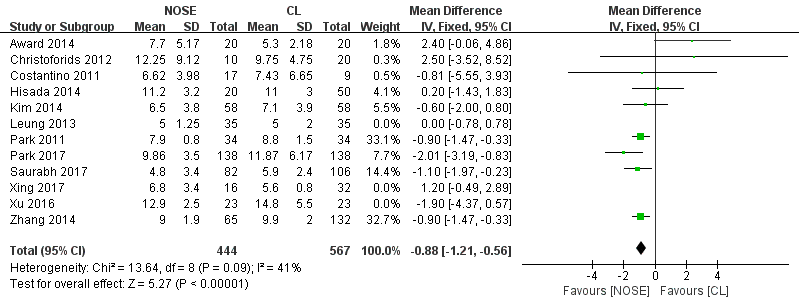


**Supplementary Figure 10D**


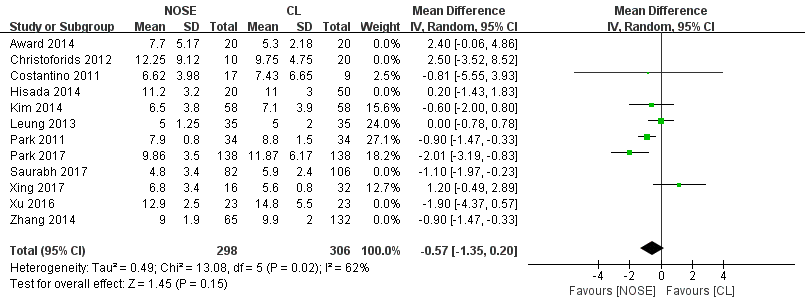

Supplement: Supplementary file 1 [file Data_Sheet_1.docx]
